# Nanoimprint lithography for nanodevice fabrication

**DOI:** 10.1186/s40580-016-0081-y

**Published:** 2016-09-01

**Authors:** Steven Barcelo, Zhiyong Li

**Affiliations:** Hewlett Packard Laboratories, 1501 Page Mill Road, Palo Alto, CA 94304 USA

**Keywords:** PDMS, Sapphire Substrate, Microlens Array, Nanoscale Device, Nanoimprint Lithography

## Abstract

Nanoimprint lithography (NIL) is a compelling technique for low cost nanoscale device fabrication. The precise and repeatable replication of nanoscale patterns from a single high resolution patterning step makes the NIL technique much more versatile than other expensive techniques such as e-beam or even helium ion beam lithography. Furthermore, the use of mechanical deformation during the NIL process enables grayscale lithography with only a single patterning step, not achievable with any other conventional lithography techniques. These strengths enable the fabrication of unique nanoscale devices by NIL for a variety of applications including optics, plasmonics and even biotechnology. Recent advances in throughput and yield in NIL processes demonstrate the potential of being adopted for mainstream semiconductor device fabrication as well.

## Introduction

Nanoimprint lithography was first defined as a nanoscale fabrication process with the potential for low cost and high throughput more than 20 years ago [1]. In the years since it has developed into a legitimate alternative to conventional lithographic technology and has found applications in many areas of nanoscale device fabrication ranging from more standard semiconductor devices [2, 3] and bit patterned media [4, 5] to more unique applications in optics [6–10] plasmonics [11–14] microfluidics [15–17] and biomimetic structures [18, 19].

Nanoimprint lithography can be defined as the use of a mold to define nanoscale deformation of a resist, which is then cured either by heat or UV application. Once the resist is cured the mold is removed and the patterned resist can be used as is or treated through etching, metal deposition or other standard lithographic techniques to generate either a final device or a new mold for further processing. The technique is broadly separated into two categories, hard and soft nanoimprint lithography. In this context, hard and soft refer to the qualities of the mold. Hard nanoimprint lithography employs a mold made of a rigid material such as silicon or quartz, enabling the support of fine features as small as 5 nm [20]. This high resolution comes at the cost of higher defect rates from particles and trapped air bubbles on a nonconforming mold and substrate. To address this challenge, the concept of soft lithography was extended to nanoimprint lithography through the use of molds composed of elastomeric material such as Poly(dimethylsiloxanes) (PDMS), polyimides and polyurethanes, although PDMS is by far the most common material [21]. Elastomeric materials can either be used as a freestanding molds for applications on curved or rough surfaces, or attached to a rigid substrate for improved durability. However, due to the higher flexibility and thermal expansion of elastomeric materials such as PDMS, registration to existing patterns can be challenging leading to overlay errors. Soft nanoimprint lithography has been further extended to roll-to-roll nanoimprint lithography for extremely high volume, low cost applications [22]. In roll-to-roll processes, typically a soft polymer mold is wrapped around a rigid roller and imprinted onto another flexible polymer web which is continuously fed through a series of rollers, yielding extremely high throughput. This complicated process leads to significant materials constraints. Molds must be flexible enough to wrap around a roller but stiff enough to emboss a resin onto a flexible substrate and have exceptional antisticking properties to allow rapid mold release after curing. Resists must spread quickly and uniformly, cure quickly and have minimal shrinkage. Despite these challenges, the promise of continuous processing has created significant interest in the field and demonstrations of nanostructured devices by roll-to-roll nanoimprint lithography are abundant.

In this review we will discuss a number of areas in the broad categories of electronics, optics and plasmonics in which NIL has been used to advance the state of the art for fabrication of nanodevices. In many cases, this is achieved by reducing the cost of fabrication either through simplified process flows or by replacing expensive lithography equipment. In other applications, unique devices are demonstrated by NIL which cannot be fabricated on a large scale by any other conventional lithography technique.

## Review

### Electronic devices

The semiconductor industry has the most stringent process constraints due to the need for high throughput, high precision extreme nanoscale fabrication of devices with low failure rates. To date, photolithography has been able to meet the demand for ever smaller devices despite the constraints imposed by the diffraction limit and the expense of deep and extreme UV light sources. However, as device sizes continue to shrink, alternative lithographic technologies, including nanoimprint lithography, must be considered if the current trends are to continue at their rapid pace. Step and repeat schemes have demonstrated the most success in maintaining the high resolution of hard masks while still enabling large area fabrication at high throughput with low defectivity [23]. Recently, a nanoimprint tool was demonstrated capable of producing more than 40 300 mm wafers per hour with a defectivity rate of less than 9 pcs/cm^2^ with a downward trend indicating that such tools may soon be competitive with extreme UV systems [24].

While nanoimprint lithography shows promise for low cost large scale nanofabrication in the future, it has played a significant role in electronic device research for many years already. Perhaps the most compelling example of this is the physical realization of the memristor, a fundamental circuit element first proposed by Chua in 1971 [25]. It was not until 2008 that experiments enabled by nanoimprint lithography were able to demonstrate the link between the coupled electronic and ionic behavior observed in nanoscale metal oxide devices and this original memristor theory [26, 27]. The experiments that led to this discovery were based on crossbar devices fabricated by nanoimprint lithography with features as small as 17 nm [28, 29]. These initial devices were based on molecular resistance switching, where nanoimprint lithography was shown to improve the performance of nanoscale devices [30]. This process was further improved with the implementation of UV NIL, reducing the temperature and pressure from thermal NIL [31]. The use of NIL not only demonstrated the potential for dense crossbar memory circuits based on nanoscale devices, but enabled inexpensive experiments and rapid turnaround for the study of material systems at an appropriate length scale. This experimental flexibility is what eventually led to a deeper understanding of the underlying physics of metal oxide resistive switching and the connection to the earlier memristor analytical models [27].

**Fig. 1:**
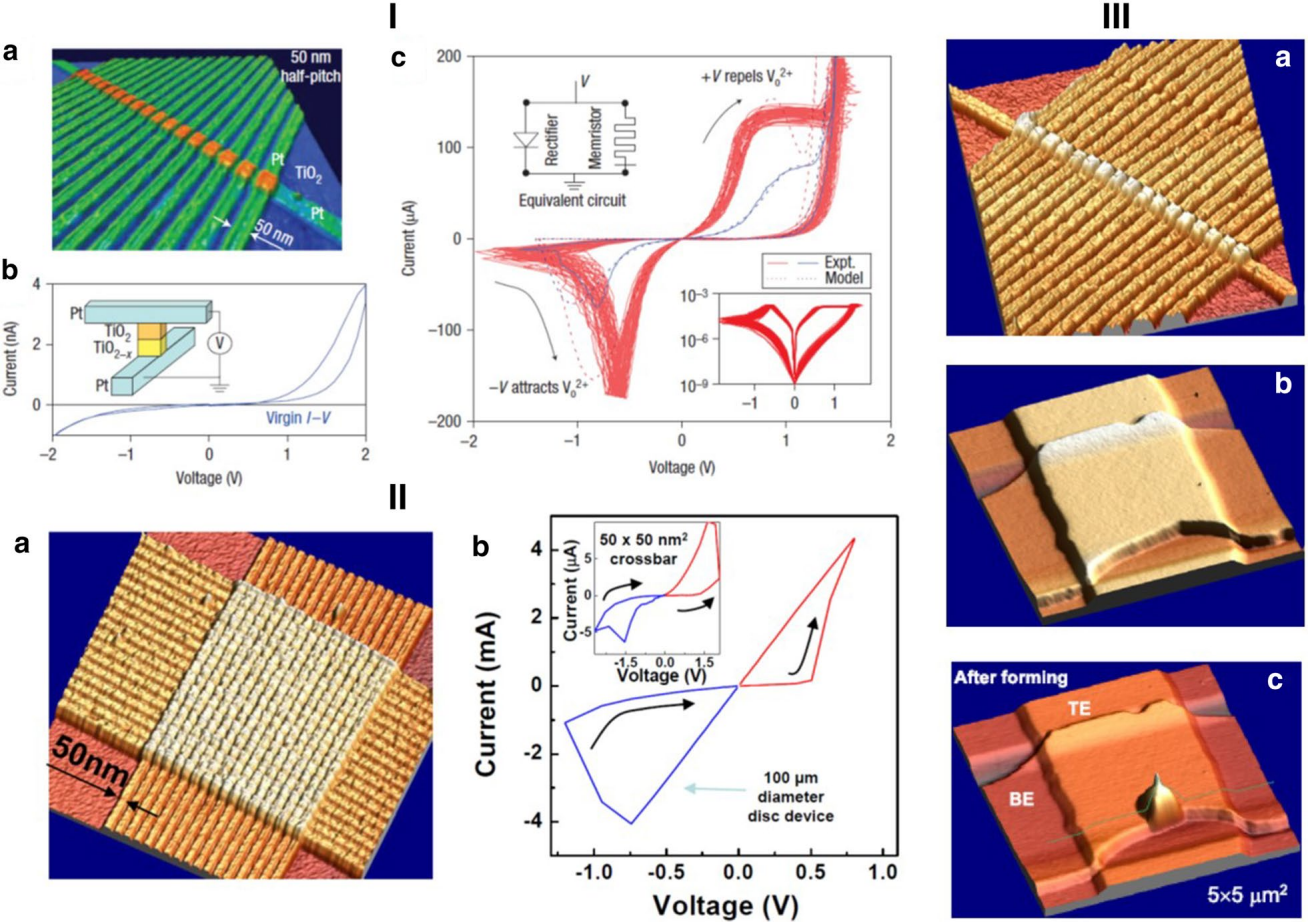
Examples of insights gained from samples fabricated by nanoimprint lithography including (*I*) devices demonstrating the mechanism behind memristive behavior in metal-oxide-metal devices showing **a** an AFM image of metal-oxide-metal devices, **b** a schematic of the device and its virgin I–V curve and **c** the circuit model for the device and its switching behavior [26]. *II* Device array used to determine the switching mechanism of TaOx based memristive devices including **a** AFM image of the 50 × 50 nm crossbar array and **b** I–V cures for the microscale and nanoscale devices validation the proposed model [32]. *III* Nanoscale and microscale memristive devices illustrate mechanism of electroforming and ways to mitigate oxygen bubble formation including AFM images of **a** nanoscale crossbar array made by nanoimprint lithography and microscale devices **b** before and **c** after electroforming showing the formation of a *bubble* near the filament in the microscale devices only [33]

Figure 1 outlines the various ways in which nanoscale patterning enabled by nanoimprint lithography furthered the understanding of memristor behavior. Panel I shows atomic force microscope (AFM) images of the first memristors, consisting of a bilayer titanium oxide (TiOx) film sandwiched between Pt electrodes, as well as the electronic performance of these devices which was shown to fit the memristor model proposed by Leon Chua [26]. Panel II shows AFM data of a nanoscale crossbar array or memristors using tantalum oxide (TaOx) as the switching material. The relative switching behavior of microscale and nanoscale devices, proving that it was possible to scale promising material systems to relevant sizes [32]. Panel III shows a nanocrossbar array with no physical deformation after switching and corresponding microscale devices with large bubble formation which led to poor reproducibility indicating that in some cases nanoscale devices could have improved performance [33].

**Fig. 2:**
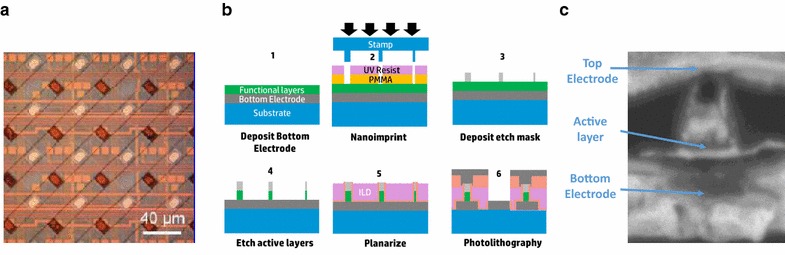
**a** Optical image of memristors integrated on *top* of CMOS circuitry [34]. **b** Process flow for the fabrication of etched bit cells by nanoimprint lithography consisting of 6 steps: *1* bottom electrode and functional layers consisting of memristor and/or selector materials are deposited. *2* Bits are patterned by nanoimprint lithography. *3* Etch mask is deposited in a lift off process. *4* Bits are created by etching through the active layers. *5* A planarization layer is deposited which also serves as an interlayer dielectric (*ILD*) which is etched back to expose the bits. *6* *Top* electrodes are patterned by photolithography. **c** SEM image of a 30 nm memristor bit fabricated by the process defined in **b**

Once the underlying physics was established, enabling the construction of models to estimate device performance, the next step was to demonstrate feasible applications of the technology. In one case, the compatibility of nanoimprint lithography with conventional semiconductor process was demonstrated by integrating a memristor array onto a complementary metal–oxide–semiconductor (CMOS) chip, as shown in Fig. 2a. This showed the potential for a chip with reconfigurable logic circuits similar to a field-programmable gate array (FPGA), but with dramatically increased density due to the promising scaling behavior of memristors [34]. More recently the investigation of more structures beyond simple crossbars have been investigated by nanoimprint lithography. Concerns about leakage current in large arrays have led to the development of more complicated material systems incorporating selector layers to reduce current leakage through unselected devices. Selector materials are often separated from the memristor by a conductive layer, requiring a patterned bit at each crosspoint. To address this, the nanoimprint process shown in Fig. 2b, c was developed capable of producing bits down to 30 nm for the study of complex memristor plus selector material stacks.

**Fig. 3:**
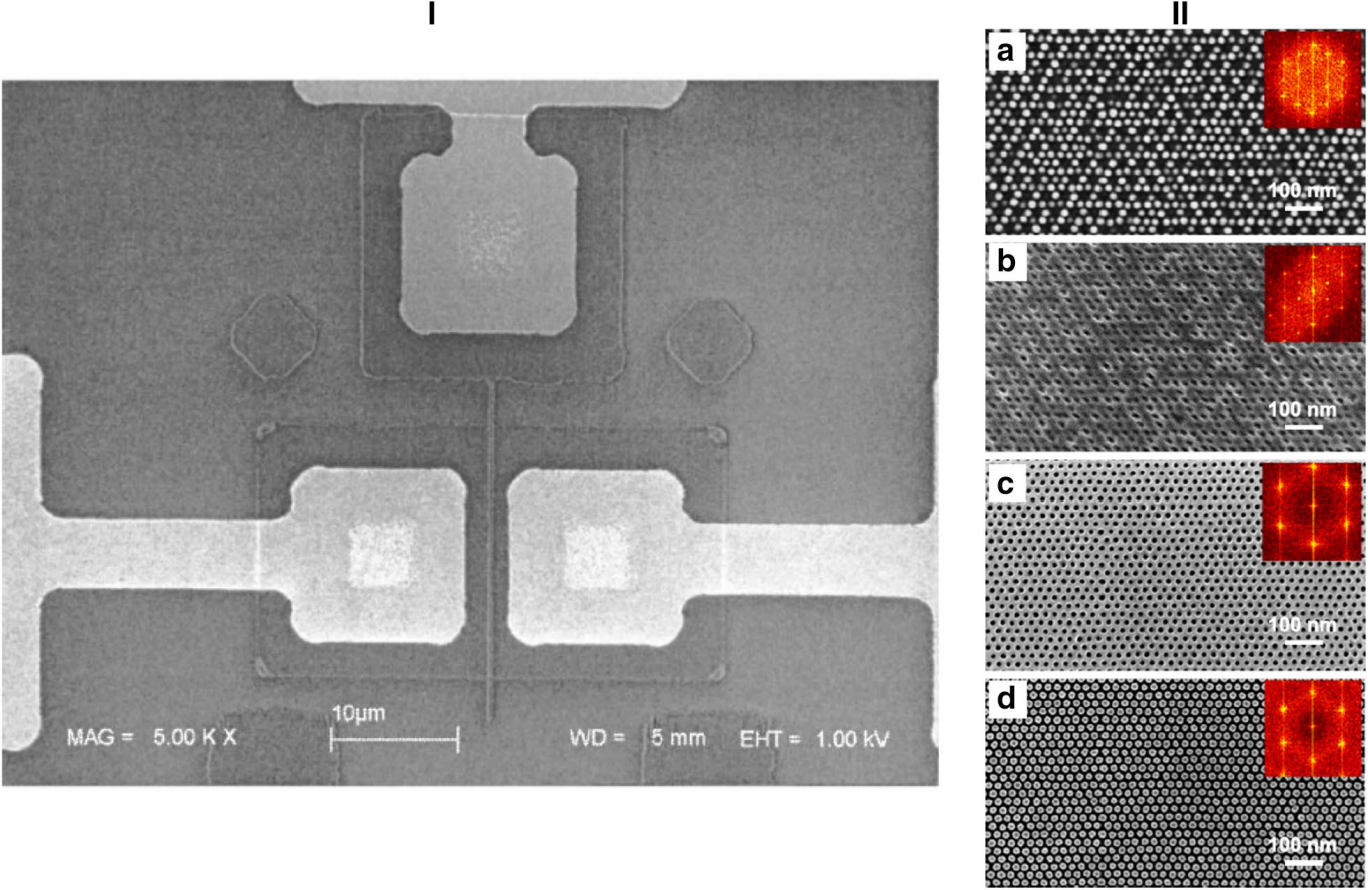
*I* SEM image of the first transistor fabricated by nanoimprint lithography [2]. *II* SEM images of the process flow to generate high fidelity nanoimprint molds for bit patterned media showing **a** the original quartz template fabricated from a sparser nanoimprint mold through a densifying DSA process showing many missing dots, **b** the resist pattern after imprint with the defective stamp, **c** the block copolymer pattern formed on *top* of the defective template and **d** the new quartz template fabricated using the BCP pattern shown in **c**. *Insets* are fast Fourier transforms of the images [5]

The fabrication of transistors by nanoimprint lithography was another one of the early indications of the promise of the technique for applications in the semiconductor industry. The first demonstration of a transistor fabricated entirely by nanoimprint lithography is shown in Fig. 3I [2]. However, given the challenges of displacing the entrenched technology of photolithography for the fabrication of high resolution transistors, the other strengths of nanoimprint lithography have often been used to address applications of transistors beyond CMOS. For example, the 3D nature of nanoimprint lithography has been used to fabricate transistors in as little as one imprint step, greatly simplifying the process and reducing cost [3]. High volume production through roll-to-roll imprinting is another extension enabling low cost transistors capable of addressing the needs of the booming flexible electronics market [35].

Bit patterned media is another field where NIL has shown great potential for large scale fabrication and was one of the first targeted applications [4]. As compared to semiconductor devices, bit patterned media requires higher resolution with slightly relaxed constraints on defect rate. Specifically, to achieve greater than 1 Tdot/in^2^, <10 nm bits are required with a defect rate of <10^−3^, whereas the current semiconductor node is 14 nm. At these length scales, fabrication of the nanoimprint mold is a significant challenge, as standard fabrication processes will have significant defects at the required density.  Multiple groups have demonstrated the potential to reduce the defect density of nanoimprint molds through directed self-assembly (DSA) of block copolymers (BCP).  DSA is the use of a patterned template, in this case a nanoimprint mold, to guide the self-assembly of BCPs into nanopatterns with good long range order and precise size and location which can then be used to fabricate a higher quality nanoimprint mold.  One approach used a nanoimprint resist specifically chosen for its compatibility with BCPs to reduce process steps.  First, a sparse pattern was created in nanoimprint resist by NIL, reducing constraints on the initial mold fabrication.  DSA was then used to densify the pattern by a factor of 4, which is controlled by the proper selection of polymer chain length to define the lattice dimensions.  Because the new mold had a relatively high defect density, it was used as a prepattern for a second DSA process, this time with no densification, leading to a high quality final mold as shown in Fig. 3II [5].

### Optical devices

One of the first commercial applications of nanoimprint lithography has been in the fabrication of optical devices due to some applications allowing for higher defect tolerance and critical feature sizes closer to 100 nm than 10 nm. Optical devices often require dense features over large areas, making direct write processes such as e-beam lithography less favorable. Furthermore, many useful optical devices can be patterned with a single layer, eliminating the overlay constraint. Nanoscale optical devices fabricated by nanoimprint lithography include wire-grid polarizers [6, 7, 36], Bragg grating filters [37] and light emitting diodes (LEDs) with improved performance [38]. Furthermore, the potential to fabricate 3-D structures in a single step leads to efficiencies in the production of lens arrays [8, 39] or other unique structures [40]. A few specific examples of optical device fabrication by nanoimprint lithography are discussed below.

**Fig. 4:**
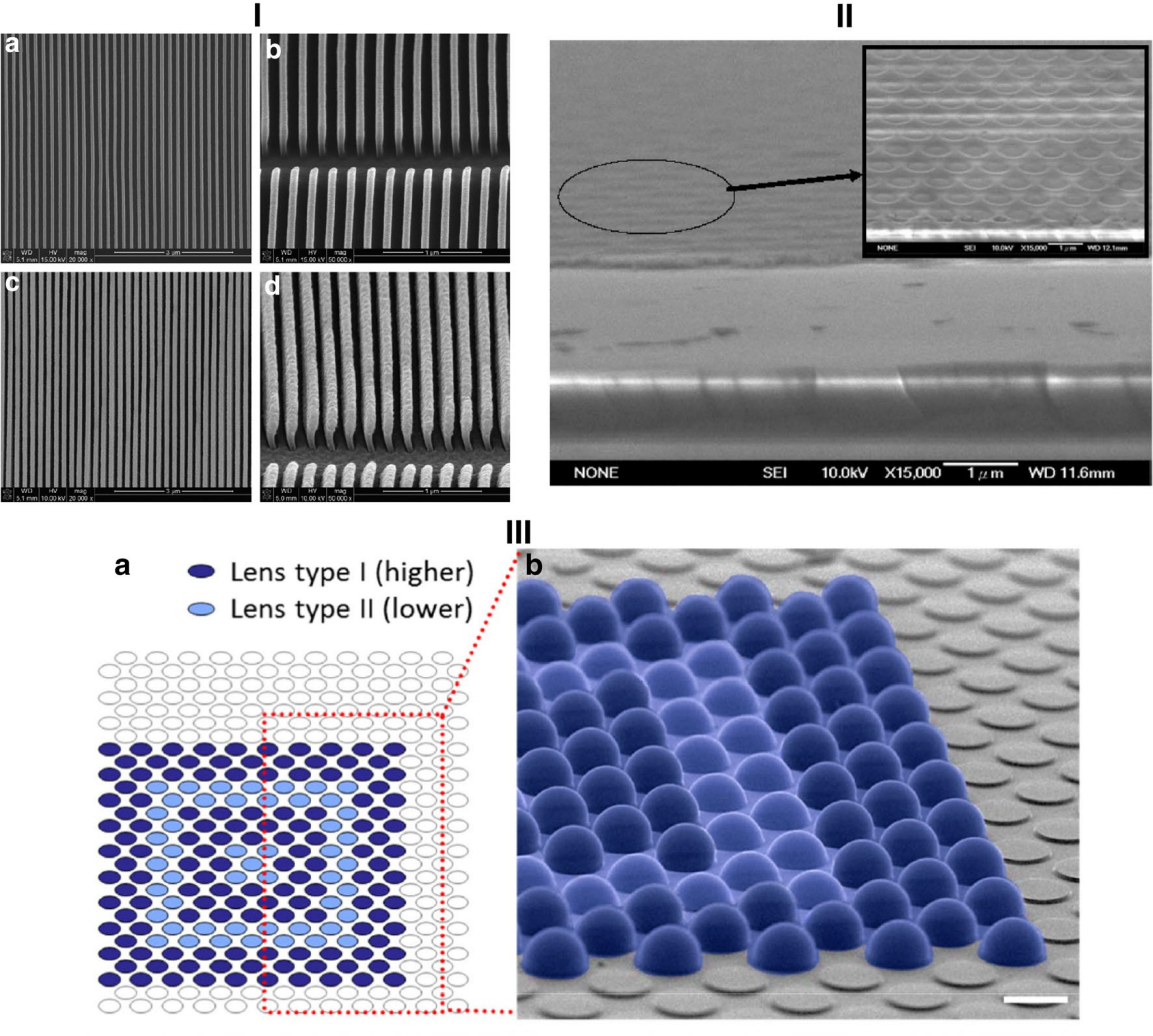
*I* SEM images of nanowire grid polarizer fabricated by nanoimprint lithography on a flexible before and after metal deposition. **a** *Top* and **b** 40^o^ *tilt view* of the imprinted grating in polycarbonate. **c** *Top* and **d** 40^o^ *tilt view* after Al deposition [7]. *II* SEM image of photonic crystal patterned onto the transparent ITO electrode of an LED by nanoimprint lithography for improved light output [38]. *III* **a** schematic and **b** SEM image of microlens arrays of varying shape patterned by nanoimprint lithography [57]

Nanowire grid polarizers consist of a subwavelength grid of metal nanowires which typically have a very large extinction ratio between the reflected and transmitted polarization and can cover a wide range of incident angle and wavelength [41]. Because of their planar structure, nanowire grid polarizers can readily be incorporated into other systems not suitable for bulk optics such as fiber-optic networks, integrated photonic circuits and liquid crystal displays [42] to name just a few. Nanoimprint lithography has been used to greatly simplify the fabrication of nanowire grid polarizers. For example, with proper material selection, nanowire grid polarizers can be made in just two steps, pattern transfer and metal deposition [9]. As shown in Fig. 4I, nanoimprint lithography has also been used to demonstrate nanowire grid polarizers on flexible substrates [7], which laid the groundwork for extremely high throughput and low cost fabrication through roll-to-roll nanoimprint lithography [43].

LEDs are an electroluminescent semiconductor light source with ubiquitous applications and a rapidly growing market. Typical length scales of LED structures range from hundreds of nanometers to a few micrometers, making it a good fit for soft nanoimprint lithography for high yield, high throughput and low cost fabrication. However, this is also a good fit for low cost optical lithography, so most nanoimprint lithography work has been on improving the performance of LEDs through the incorporation of nanoscale patterning. For example, many groups have demonstrated the integration of photonic crystals on top of LEDs by nanoimprint lithography, improving light output by as much as 25 % [10, 44, 45]. To limit etching damage, Truong et al. directly imprinted titanium oxide photonic crystals using sol gel titania as an imprint resist with a soft poly[(3-mercaptopropyl) methylsiloxane] (PMMS) mold specifically designed to allow solvent diffusion through the mold during the curing process [46]. Nanoimprint lithography has also been demonstrated as a low cost method to extend the benefits of patterned sapphire substrates to the nanoscale [47, 48]. Typically, LEDs consist of a nitride semiconductor layer on a sapphire substrate. Patterned sapphire substrates improve the efficiency of LEDs by two mechanisms. First, optimal patterning can reduce the defect density due to the lattice mismatch between the sapphire substrate and the nitride layer [49]. Second, the patterned interface scatters emitted light, reducing reflected light which is typically absorbed by the electrode [50]. Nanoscale PSS have been shown to have improved enhancement in output power over microscale PSS due to further improved scattering effects [51]. Texturing the electrode surfaces is another approach to reducing scattering losses at other interfaces within the LED structure [52]. While many approaches to electrode patterning rely on unpatterned etching, nanoimprint lithography introduces the potential for low cost pattern transfer of designed templates for optimal performance [53, 54]. One specific example of this is shown in Fig. 4II [38].

The potential to pattern 3D structures, potentially with only one process step, is a unique feature of nanoimprint lithography that can be applied to the fabrication of a variety of optical devices such as blazed gratings, microprisms and microlens arrays. Microlens arrays represent a good example for deeper consideration, since they have already achieved widespread commercialization but the new design space opened up by nanoimprint lithography could lead to many new applications. The most common approach for microlens array fabrication is thermal reflow of photoresist [55, 56]. However, this technique is limited in that the lens must be made from a thermoplastic polymer soft enough to achieve good reflow, which often are poor optical materials. While it is possible to use the photoresist as an etch mask to pattern a more desirable material, etch selectivity to a soft polymer also presents a problem. Furthermore, shape control is limited to what can be achieved using surface tension as a driving force. While it may be possible to pattern regions of varying surface energy to achieve some degree of differential lenses across a substrate, direct patterning is a much more straightforward approach. One example of the type of differential microlens array made possible by nanoimprint lithography is shown in Fig. 4III [57]. Nanoimprint lithography represents an interesting alternative due to the wide variety of materials available as imprint resists which can more readily be tailored to a particular application [8].

**Fig. 5:**
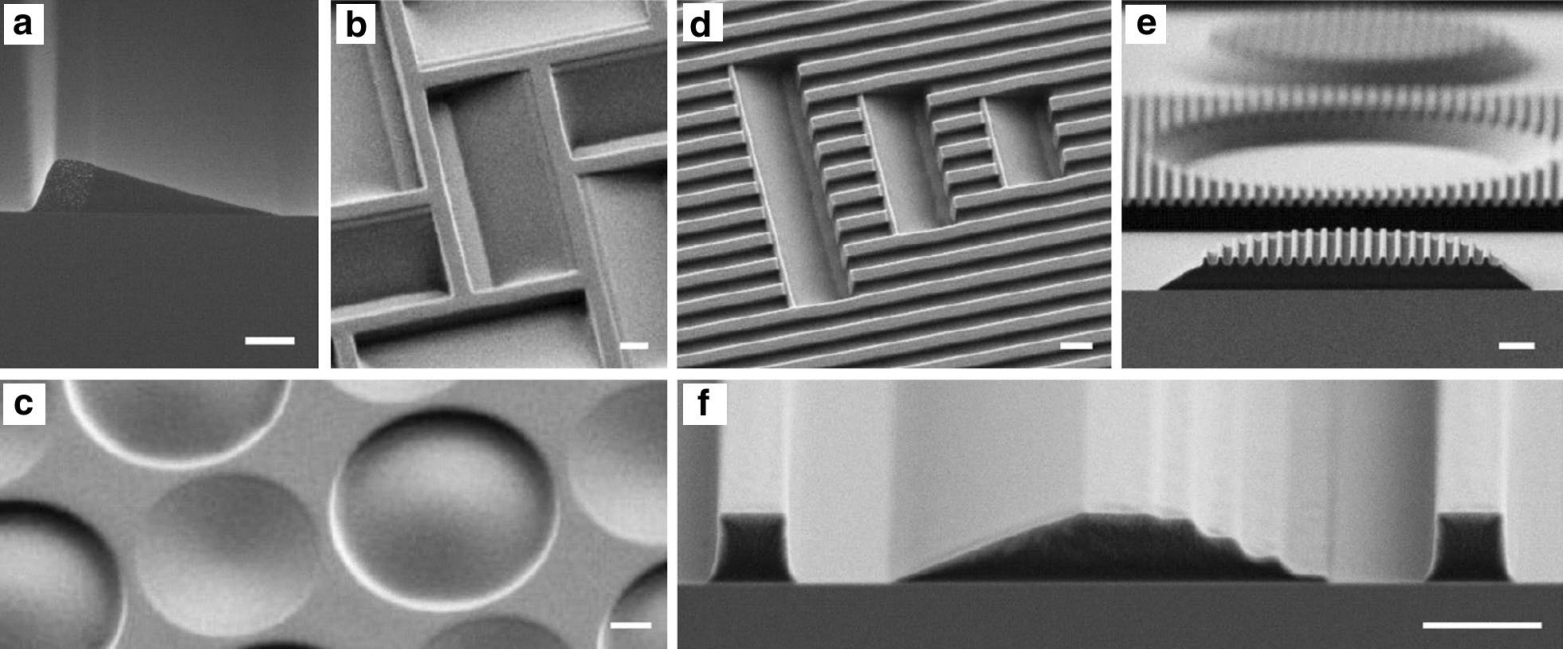
Unique 3D topographies generated by the TASTE process including **a** convex and **b** convex patterns, **c** a combination of both concave and convex patterns in close proximity, **d** *rectangular* and **e** *circular slopes* patterned into gratings and **f** a hybrid stepped and sloped structure [40]

Thermally activated selective topography equilibration (TASTE) is a new process that has been developed to take advantage of the 3D patterning potential of nanoimprint lithography for applications in both optics and fluidics [40]. The TASTE process takes advantage of the lowered T_g_ of certain polymers after irradiation to enable selective patterning of thermally induced reflow. First, a 3D topography is patterned into a polymer, typically with some form of stepped profile. Next, a specific region of the topography is irradiated to reduce its T_g_. Finally, the entire pattern is heated above the reduced T_g_ of the irradiated polymer, but below that of the unmodified polymer, leading to pattern reflow in select areas. A range of interesting contours that can be achieved by this process enabling unique grating and lens designs are shown in Fig. 5. While a variety of techniques exist which can be used to generate the initial topography, most are direct write processes such as gray scale e-beam lithography [58] or nanoscale 3D printing [59], making them more suitable for mold fabrication. Therefore thermal nanoimprint lithography is preferred for the large scale application of the TASTE process.

### Plasmonic devices

**Fig. 6:**
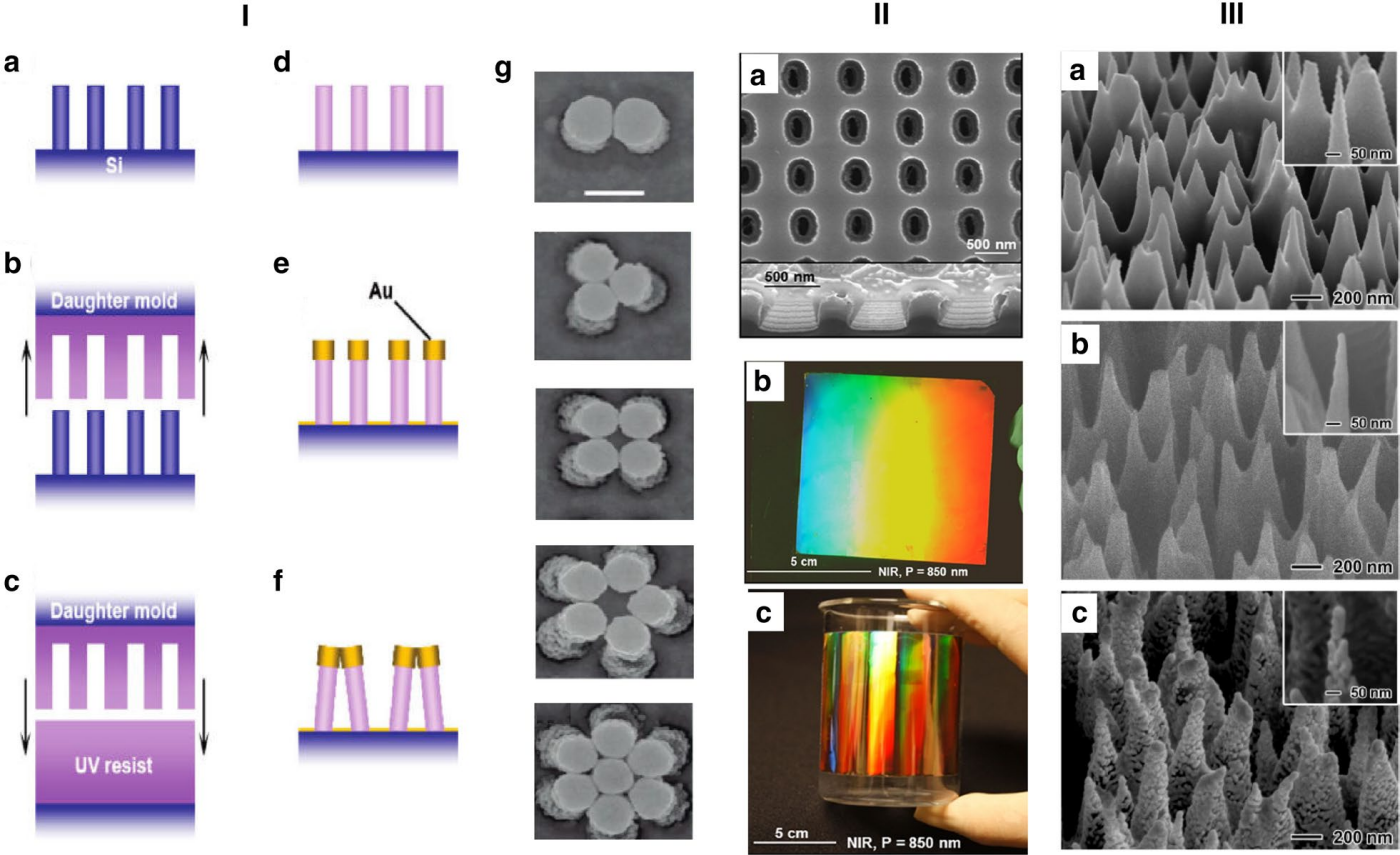
*I* Overview of the nanoimprint process for the fabrication of gold nanofingers showing **a** patterning of pillars and etching into Si master mold, **b** first imprint transferring pattern to daughter mold, **c** second imprint creating **d** original pillar pattern, **e** evaporation of Au metal to form particles on pillar tips and **f** collapsing of pillars into the designed geometries. **g** SEM images of a representative set of particle arrangements fabricated using this technique. Scale bar is 200 nm [67]. *II* Sequence of SEM images showing **a** Si cones fabricated by a Bosch etch process, **b** replicated in a polymer by nanoimprint lithography while maintaining sharp tips as required for optimal SERS performance and **c** coated with gold [61]. *III* **a** *Top* and *sideview* images of a multilayer 3D negative index material fabricated by nanoimprint lithography on both **b** a rigid and **c** flexible substrate [70]

The patterning of metallic nanoparticles or nanovoids to manipulate their plasmonic properties is another field with a wealth of applications, including nanoscale optics [11, 12], metamaterials [60, 61], and chemical and biological sensing [13, 14]. One of the most popular applications of plasmonics is surface enhanced Raman spectroscopy (SERS), where the electric field, E, generated by local surface plasmons can be used to enhance Raman scattering by a factor of |E| [4, 62]. Some of the strongest SERS enhancement has been detected at the junction of metallic nanoparticles, often with a nanoscale gap on the order of 2 nm or less [63, 64]. However, lithographic patterning of such narrow gaps is prohibitive. An alternative approach takes advantage of elasto-capillary forces to pattern groups of pillars that can be coated with an appropriate metal and collapsed into an optimal structure for SERS [65]. A simple fabrication method using maskless etching has been demonstrated to generate collapsible silicon pillars, but random placement of the pillars leads to suboptimal nanoparticle geometry [66]. Using nanoimprint lithography with a high aspect ratio mold, it is possible to directly imprint flexible polymer pillars which can be collapsed into assemblies with optimized geometry, as shown in Fig. 6I [67]. Subsequent imprint steps can then be used to transfer the nanoparticle assemblies onto other substrates [68] or into solution [69] to extend the applications.

The ability to realize 3D structures by nanoimprint lithography generates a significant design space for the fabrication of plasmonic structures. For example, negative index materials consist of multiple layers of fishnet or Swiss-cross patterns. Nanoimprint lithography has been used to demonstrate the feasibility of large scale fabrication of such devices on flexible substrates, with one example shown in Fig. 6II [60, 61]. In the field of SERS, a Si cone structure was replicated into a polymer film, critically maintaining the sharp tip required for a strong SERS signal only possible via high resolution UV nanoimprint lithography as shown in Fig. 6III [70]. Multiple groups have fabricated 3D structures with strong SERS performance such as pyramids [71] and nanohole arrays [72] to a flexible substrate, demonstrating the potential for high volume production of 3D SERS structures through roll-to-roll manufacturing.

## Conclusion

Nanoimprint lithography is a versatile technique with applications across all industries involving nanofabrication. Here we have discussed numerous examples of the impact of nanoimprinting on the fabrication of devices in the semiconductor industry, in optics and in the emerging field of plasmonics. In each case, the demands of the industry are different, but the strengths of nanoimprint lithography make it a useful tool in many instances. In the semiconductor industry nanoimprint lithography has long been a cost effective method for studying nanoscale device behavior. However, as device sizes shrink there is increasing potential to find an alternative to optical lithography, and nanoimprint lithography is one of the leading contenders. In the field of optics the low cost, simple process and material flexibility have led to numerous applications and simpler integration of the end products into more complex systems such as lab on a chip or integrated photonic circuits. In the field of plasmonics, many unique structures have been demonstrated which would be challenging or impossible to fabricate using any other technique.

## Abbreviations

NIL: nanoimprint lithography
PDMS: poly (dimethylsiloxane)
AFM: atomic force microscope
CMOS: complementary metal–oxide–semiconductor
LED: light emitting diode
PMMS: poly[(3-mercaptopropyl) methylsiloxane]
TASTE: thermally activated selective topography equilibration
